# Associations of drinking rainwater with macro-mineral intake and cardiometabolic health: a pooled cohort analysis in Bangladesh, 2016–2019

**DOI:** 10.1038/s41545-020-0067-5

**Published:** 2020-04-24

**Authors:** Abu Mohd Naser, Mahbubur Rahman, Leanne Unicomb, Sarker Masud Parvez, Shariful Islam, Solaiman Doza, Golam Kibria Khan, Kazi Matin Ahmed, Shuchi Anand, Stephen P. Luby, Mohammad Shamsudduha, Matthew O. Gribble, K. M. Venkat Narayan, Thomas F. Clasen

**Affiliations:** 1 Emory Global Diabetes Research Center, Hubert Department of Global Health, Rollins School of Public Health, Emory University, Atlanta, GA, USA; 2 Gangarosa Department of Environmental Health Sciences, Rollins School of Public Health, Emory University, Atlanta, GA, USA; 3 International Centre for Diarrhoeal Disease Research, Bangladesh (icddr,b), GPO Box 128, Dhaka 1000, Bangladesh; 4 Department of Agricultural Chemistry, Patuakhali Science and Technology University, Dumki, Patuakhali 8602, Bangladesh; 5 Department of Geology, University of Dhaka, Dhaka, Bangladesh; 6 Division of Nephrology, School of Medicine, Stanford University, Stanford, CA, USA; 7 Woods Institute for the Environment, Stanford University, Stanford, CA, USA; 8 Institute for Risk and Disaster Reduction, University College London, London, UK; 9 Department of Geography, University of Sussex, Brighton, UK; 10 Department of Epidemiology, Rollins School of Public Health, Emory University, Atlanta, GA, USA

## Abstract

This study explores the associations of drinking rainwater with mineral intake and cardiometabolic health in the Bangladeshi population. We pooled 10030 person-visit data on drinking water sources, blood pressure (BP) and 24-h urine minerals. Fasting blood glucose (FBG) was measured in 3724 person-visits, and lipids in 1118 person-visits. We measured concentrations of sodium (Na), potassium (K), calcium (Ca) and magnesium (Mg) in 253 rainwater, 935 groundwater and 130 pond water samples. We used multilevel linear or gamma regression models with participant-, household- and community-level random intercepts to estimate the associations of rainwater consumption with urine minerals and cardiometabolic biomarkers. Rainwater samples had the lowest concentrations of Na, K, Ca and Mg. Rainwater drinkers had lower urine minerals than coastal groundwater drinkers: −13.42 (95% CI: −18.27, −8.57) mmol Na/24 h, −2.00 (95% CI: −3.16, −0.85) mmol K/24 h and −0.57 (95% CI: −1.02, −0.16) mmol Mg/24 h. The ratio of median 24-hour urinary Ca for rainwater versus coastal groundwater drinkers was 0.72 (95% CI: 0.64, 0.80). Rainwater drinkers had 2.15 (95% CI: 1.02, 3.27) mm Hg higher systolic BP, 1.82 (95% CI: 1.19, 2.54) mm Hg higher diastolic BP, 0.59 (95% CI: 0.17, 1.01) mmol/L higher FBG and −2.02 (95% CI: −5.85, 0.81) mg/dl change in high-density lipoprotein cholesterol compared with the coastal groundwater drinkers. Drinking rainwater was associated with worse cardiometabolic health measures, which may be due to the lower intake of salubrious Ca, Mg and K.

## Introduction

Rainwater harvesting is currently practiced worldwide for potable and non-potable uses^1^. Rainwater-harvesting systems are important sources of water for rural or remote areas that do not have a piped water supply but have plenty of rainfall. Harvesting rainwater provides contamination-free drinking water in areas of developing countries where faecal pathogens or chemicals contaminate other drinking water sources such as surface water or groundwater^2^. The dependence of water from rainwaterharvesting systems is increasing due to progressive stress on groundwater sources and proliferation of groundwater recharge systems^3,4^, coastal seawater intrusion^5^, microbiological and chemical contamination of groundwater and surface water^6^, growing awareness of water conservation^7^ and promotion of environment-friendly housing developments^1,8^.

Groundwater is the primary source of drinking water in Bangladesh^9^. However, seawater intrusion has resulted in salinity-induced water scarcity in southwest coastal regions of Bangladesh^10^. Epidemiological studies from southwest coastal Bangladesh have found positive associations of drinking saline water with sodium (Na) intake, blood pressure, and with hypertensive disorders of pregnant women (e.g., preeclampsia) ^11–14^. To address water salinity problems, rainwater-harvesting systems and pond sand filters near rainwater-fed ponds are currently being promoted in southwest coastal Bangladesh^15,16^. These systems capture rainwater during the wet season when sufficient rainfall is available and conserve water for future use during the dry season.

Drinking rainwater may have unintended adverse effects on public health. Harvested rainwater may be contaminated by faecal indicator bacteria and pathogenic microorganisms^6^. Chemicals may leach from the roof surface, drainage gutter and storage tank^17,18^. Even in the absence of contamination, there may be adverse health implications, as the water may lack important constituents. Calcium (Ca), magnesium (Mg) and potassium (K) are essential macrominerals for humans, inadequate intake of which is associated with cardiovascular events^19–21^, and these minerals are present at low or negligible levels in rainwater. This raises concern that rainwater may have detrimental effects on cardiometabolic health of the population, compared with alternative sources of water. To explore this hypothesis, we conducted an observational epidemiology study comparing rainwater drinkers versus other water (e.g., groundwater and surface water) drinkers for biomarkers of essential macrominerals, and for cardiometabolic parameters (urine protein, blood pressure, blood glucose and blood lipids) in Bangladesh.

## Results

### Demographics of the participants

The pooled cohort included 10030 person-visits, 8261 from the coastal region and 1773 from the non-coastal region. Participants exclusively drank groundwater in the non-coastal region. Of the 8261 coastal person-visits, 1620 drank rainwater exclusively, 2836 drank pondwater only, and 3225 drank groundwater exclusively, and the others reported drinking from multiple water sources (Table 1).

The mean 24-h urinary Na was 138.5 (95% CI: 134.7, 142.3) mmol/24 h, chloride was 147.7 (95% CI: 144.2, 151.1) mmol/24 h, K was 31.8 (95% CI: 31.1, 32.6) mmol/24 h, Mg was 3.3 (95% CI: 3.2, 3.4) mmol/24 h and median Ca was 2.7 (IQR: 1.6–4.2) mmol/24 h among the person-visits of rainwater drinkers (Table 2). The mean 24-h urinary Na was 166.5 (95% CI: 164.0, 169.2) mmol/24 h, chloride was 175.4 (95% CI: 172.6, 178.3) mmol/24 h, K was 33.3 (95% CI: 32.8, 33.9) mmol/24 h, Mg was 4.0 (95% CI: 3.9, 4.1) mmol/ 24 h and median Ca was 3.6 (IQR: 2.0–5.9) mmol/24 h among the person-visits of coastal groundwater drinkers (Table 2). Person-visits of rainwater drinkers had mean 114.18 (95% CI: 113.39, 114.97) mm Hg systolic blood pressure (SBP), 68.29 (95% CI: 67.79, 68.79) mm Hg diastolic blood pressure (DBP), 6.64 (95% CI: 6.46, 6.81) mmol/L fasting blood glucose (FBG), 34.11 (95% CI: 32.56, 35.65) mg/dl high-density lipoprotein cholesterol (HDL-C) and median 173.95 (IQR: 98.60–278.92) mg/24-h urine total protein. Person-visits of coastal groundwater drinkers had mean 111.21 (95% CI: 110.67, 111.75) mm Hg SBP, 65.97 (95% CI: 65.62, 66.32) mm Hg DBP, 5.33 (95% CI: 5.20, 5.46) mmol/L FBG, 36.24 (35.48, 37.01) mg/dl HDL-C and median 188.70 (IQR: 111.40–294.45) mg/ 24-hour urine total protein (Table 2).

### Water mineral concentrations in different water sources

**Fig. 1:**
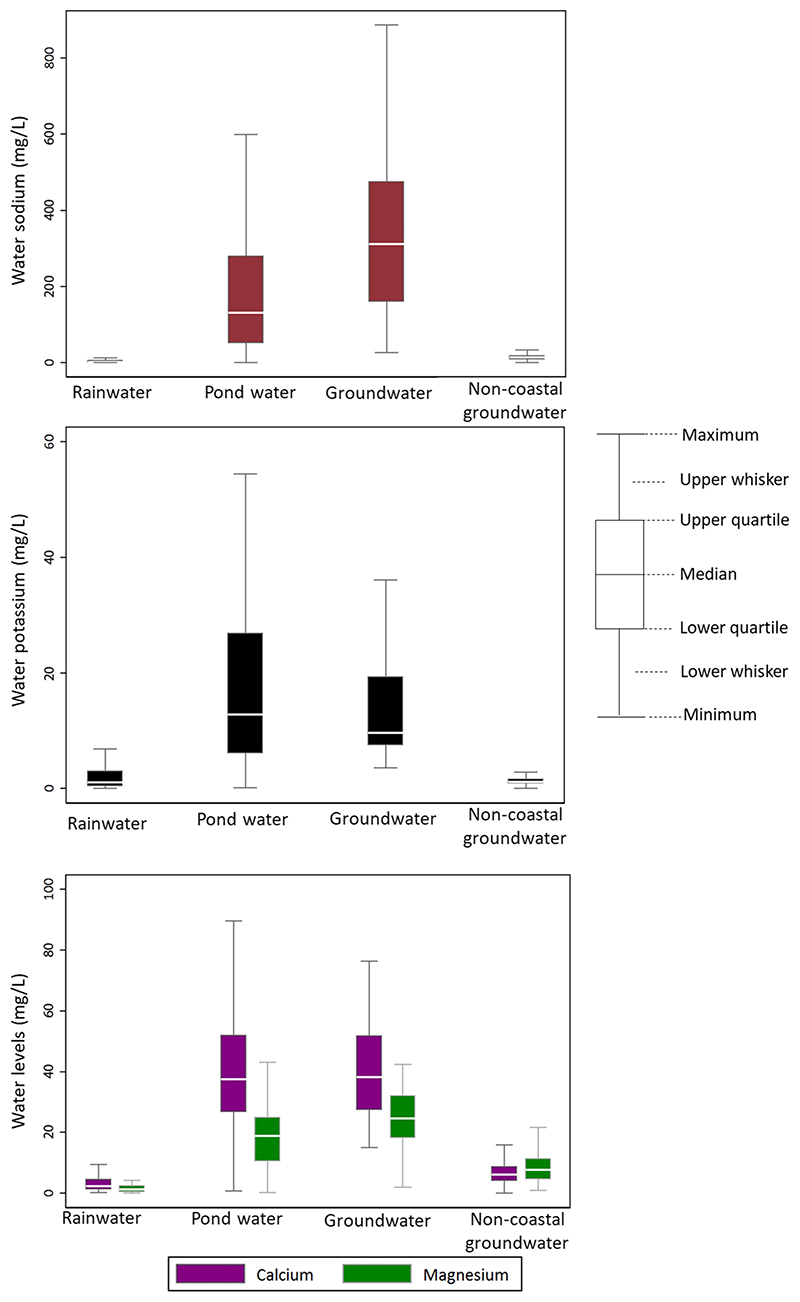
Concentrations of sodium, potassium, calcium and magnesium in different water sources. This figure suggests rainwater has deficient concentrations of sodium, potassium, calcium, and magnesium.

We collected 253 rainwater, 130 pondwater, 45 coastal ground-water and 890 non-coastal groundwater samples. The median Na and K concentrations were 2.36 (IQR: 0.97–5.55) mg/L and 1.10 (IQR: 0.38–3.02) mg/L in rainwater, 130.71 (IQR: 51.95–280.29) mg/ L and 12.80 (IQR: 6.08–26.84) mg/L in pond water, 311.64 (IQR: 159.72–474.76) mg/L and 9.67 (IQR: 7.46–19.33) mg/L in coastal groundwater and 13.95 (IQR: 9.38–19.04) mg/L and 1.14 (IQR: 0.87–1.65) mg/L in non-coastal groundwater (Fig. 1). The median Ca and Mg concentrations were 2.30 (IQR: 1.21–4.59) mg/L and 1.21 (IQR: 0.47–2.50) mg/L in rainwater, 37.57 (IQR: 26.64–51.99) mg/L and 18.82 (IQR: 10.46–25.01) mg/L in pondwater, 38.27 (IQR: 27.45–51.82) mg/L and 24.64 (IQR: 18.23–32.11) mg/L in coastal groundwater and 6.19 (IQR: 4.00–8.80) mg/L and 7.76 (IQR: 4.55–11.40) mg/L in non-coastal groundwater (Fig. 1).

### Twenty-four-hour intake of minerals among rainwater drinkers

Compared with person-visits of coastal groundwater drinkers, those among rainwater drinkers had lower urine minerals: −13.42 (95% CI: −18.27, −8.57) mmol/24 h urinary Na, −13.44 (95% CI: −19.21, −7.66) mmol/24 h urinary chloride, −2.00 (95% CI: −3.16, −0.85) mmol/24 h urinary K and −0.57 (95% CI: −1.02, −0.16) mmol/24 h urinary Mg in the fully adjusted models (Table 3). The ratio of median 24 h urinary Ca for person-visit of rainwater drinkers versus those in coastal groundwater drinkers was 0.72 (95% CI: 0.64, 0.80) in the fully adjusted model (Table 3). The associations of the water source with urine Na, chloride, K, Ca and Mg were Bonferroni-significant. When rainwater drinkers were compared with both coastal and non-coastal groundwater drinkers combined, the estimates for urinary Na and Mg were attenuated (Table 4).

Compared with person-visits of pond water drinkers, those among rainwater drinkers had lower urine minerals: −12.09 (95% CI: −18.50, −5.68) mmol/24 h urinary Na, −12.95 (95% CI: −19.82, −6.09) mmol/24 h urinary chloride, −2.23 (95% CI: −3.92, −0.53) mmol/24 h urinary K and −0.17 (95% CI: −0.45, 0.11) mmol/24 h urinary Mg in fully adjusted models (Table 5). The ratio of median 24 h urinary Ca for person-visit of rainwater drinkers versus those in pond water drinkers was 0.86 [95% CI: 0.79, 0.93] in the fully adjusted model (Table 5).

### Cardiovascular biomarkers among rainwater drinkers

Compared with person-visits of coastal groundwater drinkers, those among rainwater drinkers had 2.15 (95% CI: 1.02, 3.27) mm Hg higher SBP, 1.82 (95% CI: 1.19, 2.45) mm Hg higher DBP, 0.59 (95% CI: 0.17, 1.01) mmol/L higher FBG, −2.02 (95% CI: −5.85, 0.81) mg/dl change in HDL-C and −2.30 (95% CI: −12.92, 8.32) mg/ dl change in total cholesterol in fully adjusted models (Table 3). The ratio of median 24-hour urine protein was 1.16 (95% CI: 1.01, 1.33), and median triglycerides were 1.06 (95% CI: 0.89, 1.25) for person-visits of rainwater versus those in coastal groundwater drinkers in the fully adjusted models (Table 3). The association of rainwater consumption with higher SBP, DBP and FBG was Bonferroni-significant. When rainwater drinkers were compared with both coastal and non-coastal groundwater drinkers combined, the estimates for cardiometabolic biomarkers were similar (Table 4).

Compared with person-visits of pond water drinkers, those among rainwater drinkers had 0.91 [95% CI: −0.54, 2.36] mm Hg change in SBP, 0.53 [95% CI: −0.39, 1.44] mm Hg change in DBP, −0.04 (95% CI: −0.21, 0.12) mmol/L increase in FBG, −3.40 (95% CI: −6.69, −0.11) mg/dl change in HDL-C and −1.18 (95% CI: −15.19, 12.83) mg/dl change in total cholesterol in fully adjusted models (Table 5). The ratio of median 24-hour urine protein was 0.89 [95% CI: 0.74, 1.07] and median triglyceride was 1.10 [95% CI: 0.89, 1.35] for person-visits of rainwater versus those in pond water drinkers in the fully adjusted models (Table 5).

In propensity-score-matched analyses, compared with person-visits of coastal groundwater drinkers, those among rainwater drinkers had 1.74 (95% CI: 0.71, 2.76) mm Hg higher SBP, 1.61 (95% CI: 1.03, 2.19) mm Hg higher DBP, 0.59 (95% CI: 0.17, 1.01) mmol/L higher FBG and −3.25 (95% CI: −6.35, −0.15) mg/dl change in HDL-C (Table 6). The ratio of median 24-h urine protein was 1.17 (95% CI: 1.02, 1.33) for person-visits of rainwater versus those in coastal groundwater drinkers in the fully adjusted models (Table 6). Similarly, in propensity-score-matched analyses, compared with person-visits of pond water drinkers, those among rainwater drinkers had 0.81 (95% CI: −0.42, 2.03) mm Hg change in SBP, 0.35 (95% CI: −0.58, 1.28) mm Hg change in DBP, −0.04 (95% CI: −0.22, 0.14) mmol/L change in FBG and −3.29 (95% CI: −7.08, 0.51) mg/ dl change in HDL-C (Table 6). The ratio of median 24-hour urine protein was 0.92 (95% CI: 0.76, 1.10) for person-visits of rainwater versus those in pond water drinkers in the fully adjusted models (Table 6).

### Sensitivity analyses

In conditional logistic regression analyses for within-person comparison of drinking rainwater versus coastal groundwater, we found that drinking rainwater was associated with lower urinary K (*P*-value: <0.001), Ca (*P*-value: <0.001) and Mg (*P*-value: <0.001). We also found that drinking rainwater was associated with a higher SBP (*P*-value: <0.001), DBP (*P*-value: <0.001) and FBG (*P*-value: <0.001) (Table 7).

## Discussion

We found that rainwater has deficient concentrations of minerals, and drinking rainwater is associated with lower daily intakes of all essential macrominerals. Concentrations of several biomarkers suggesting drinking rainwater compared with drinking ground-water may increase the risk of cardiometabolic diseases.

Rainwater had a lower concentration of Na, and a low level of Ca, Mg and K compared with groundwater or pondwater. Pondwater in the coastal region had almost a similar concentration of Ca and K, and a slightly reduced concentration of Na and Mg compared with the coastal groundwater. Another study in southwest coastal Bangladesh also noticed similar findings^22^. The mean Ca concentrations were 0.9 mg/L for rainwater, 116 mg/L for groundwater and 50 mg/L for pondwater; the mean Mg concentration was 0.08 mg/L for rainwater, 95 mg/L for groundwater and 32 mg/L for pondwater; the mean K concentration was 0.1 mg/L for rainwater, 25 mg/L for groundwater and 15 mg/L for pond-water^22^. Secondary analysis of groundwater chemistry data from the National Hydrochemical Survey conducted jointly by the British Geological Survey (BGS), and Department of Public Health Engineering (DPHE) suggests that individuals can obtain up to half of the dietary reference intake (DRI) of Ca and Mg by drinking 2 L of groundwater in some areas of Bangladesh^23^.

We found that rainwater drinkers had a lower urinary level of Na, chloride, Ca, Mg and K than the coastal groundwater or pond water drinkers in all models. Readily absorbable ionic forms of minerals in drinking water facilitate the higher absorption of minerals from drinking water^24^. Na and chloride are the extracellular ions for humans; therefore, their concentrations in urine and blood are higher than those of K, Ca and Mg.

The drinking water sources in coastal Bangladesh vary across the season. Rainwater consumption in coastal Bangladesh is determined by rainfall or availability of the stored harvested rainwater. Consumption of rainwater compared with groundwater or pondwater as a potable water source in coastal Bangladesh reduced exposure to harmful Na and likely benefited the cardiovascular health of the population. Nevertheless, such benefit will not be substantial since, despite drinking rainwater, the mean daily Na intake of this population was 139 mmol/24 hours, which is much higher than the WHO-recommended 87 mmol/24 h^25^. This indicates that the coastal Bangladeshi population had much higher Na intake through food than drinking water. Unless Na intake reduction interventions from the diet are accompanied by Na intake reduction strategies from the drinking water, the cardiovascular health benefits will be trivial.

In contrast, consumption of rainwater compared with consumption of coastal groundwater also reduced intakes of cardioprotective Ca, Mg and K in this study population. Although drinking water is not the primary source of these salubrious minerals, it may serve as an important contributor when regular diets, especially in low-resource environments, have low or borderline intakes in these elements^24^. The global population has low intakes of Ca, Mg and K^26,27^—more than 50% population in the United States have lower intakes Ca and Mg than the daily recommended intake^28^. Dietary surveys among the Bangladeshi population suggest that the intake of Ca is low through the available food system of households^29,30^. Therefore, the promotion of rainwater risks further reducing mineral intakes in this mineral-deficient population.

Our study found that drinking rainwater was associated with higher SBP, DBP, FBG, urinary protein and decreased HDL-C compared with coastal groundwater drinkers at the nominal significance P-value threshold of 0.05—all these biomarkers are suggestive of increased cardiometabolic disease risks. Following Bonferroni-corrected P-value threshold of 0.01, rainwater consumption was associated with higher SBP, DBP and FBG. These findings suggest despite a substantial lower intake of Na, rainwater drinkers had higher cardiometabolic disease risks compared with coastal groundwater drinkers.

The higher cardiometabolic disease risks among the rainwater drinkers compared with coastal groundwater are likely due to reduced intake of Ca, Mg and K. We elsewhere reported that groundwater minerals influence blood pressure of the Bangladeshi population^31^, and the blood pressure-lowering effects of Ca and Mg from drinking water overweighed the blood pressurepromoting effects of Na^32^, which explains the lower blood pressure among groundwater drinkers compared with rainwater drinkers. The findings from this study have likely generalizable implications in other saline-water-affected coastal regions, where rainwater and desalinated water without mineral fortification are being promoted to mitigate water salinity problems. The predominant cations in saline water are Na, Ca and Mg across seawater intrusion-affected coastal regions^23,33–35^. When rainwater without any mineral fortification will be promoted to mitigate water salinity, communities will likely have higher cardiometabolic disease risk, as evident by our findings. Higher cardiovascular disease prevalence and mortality were observed among hospitalized patients in desalination plant-served regions of Israel^36,37^—patients in desalinated areas had lower intakes of Mg than patients in non-desalinated areas.

Our analyses have several limitations. We adjusted for several known confounders of cardiometabolic diseases, but the unmeasured confounders may have influenced our estimates. To overcome this, we tried two approaches of matched analyses to reduce the influence of different confounders, and we found similar conclusions from the matched analyses. The coastal and non-coastal communities seemed different in many aspects. However, our estimates did not change when the non-coastal groundwater drinkers were excluded in analyses. Weather variables such as temperature, rainfall and humidity might also influence blood pressure outcomes^38–40^—and we were unable to control for it. Nevertheless, adjusting the models with the season may have lowered such bias. Blood pressure of an individual has a diurnal variation^41^—we did not collect the accurate time of blood pressure measurement and were thereby unable to control for it. Although 24-h urine collection is the gold-standard method for daily Na intake, such day-long urine collection may be biased by over- or under-collection of urine samples^42^. Several studies have reported Na^+^-induced Ca excretion through urine^43^—therefore, high urinary Ca among groundwater and pond water drinkers could be partially due to the influence of Na on kidneys. However, high Ca concentrations in groundwater, and pond water sources suggest that such findings are not by chance.

Currently, no guideline exists about the minimum level of salubrious minerals in drinking water. WHO has not set up such a cut-off point of mineral concentrations in drinking water considering human health^44^. Nevertheless, WHO has highlighted the public health significance of calcium and magnesium in drinking water^45^, and suggested that the local public health and nutrition experts need to decide whether the low salinity (e.g., desalinated water) needs mineral fortification of drinking water^46^. Adding salubrious minerals to drinking water may be a useful strategy for reducing the population burden of cardiometabolic disease risks when drinking water sources have low levels of these minerals since concentrations of these minerals are decreasing in the diet of the population globally^26,27^. In line with WHO recommendation of desalination water^46^, public health and nutrition experts in southwest coastal Bangladesh need to consider adding minerals in rainwater for public health safety. The efficacy of such mineral fortification of rainwater needs to be evaluated through epidemiological studies. Widespread promotion of rainwater by the public- and private-sector partners, and water practitioners in coastal Bangladesh to lower Na intake through drinking water will otherwise put the communities in higher cardiometabolic disease risks. If salubrious minerals are not added to the rainwater, the adverse health consequences of drinking rainwater will likely be undetected since no epidemiological surveillance systems currently exist in coastal Bangladesh.

## Methods

### Data sources and study settings

**Fig. 2:**
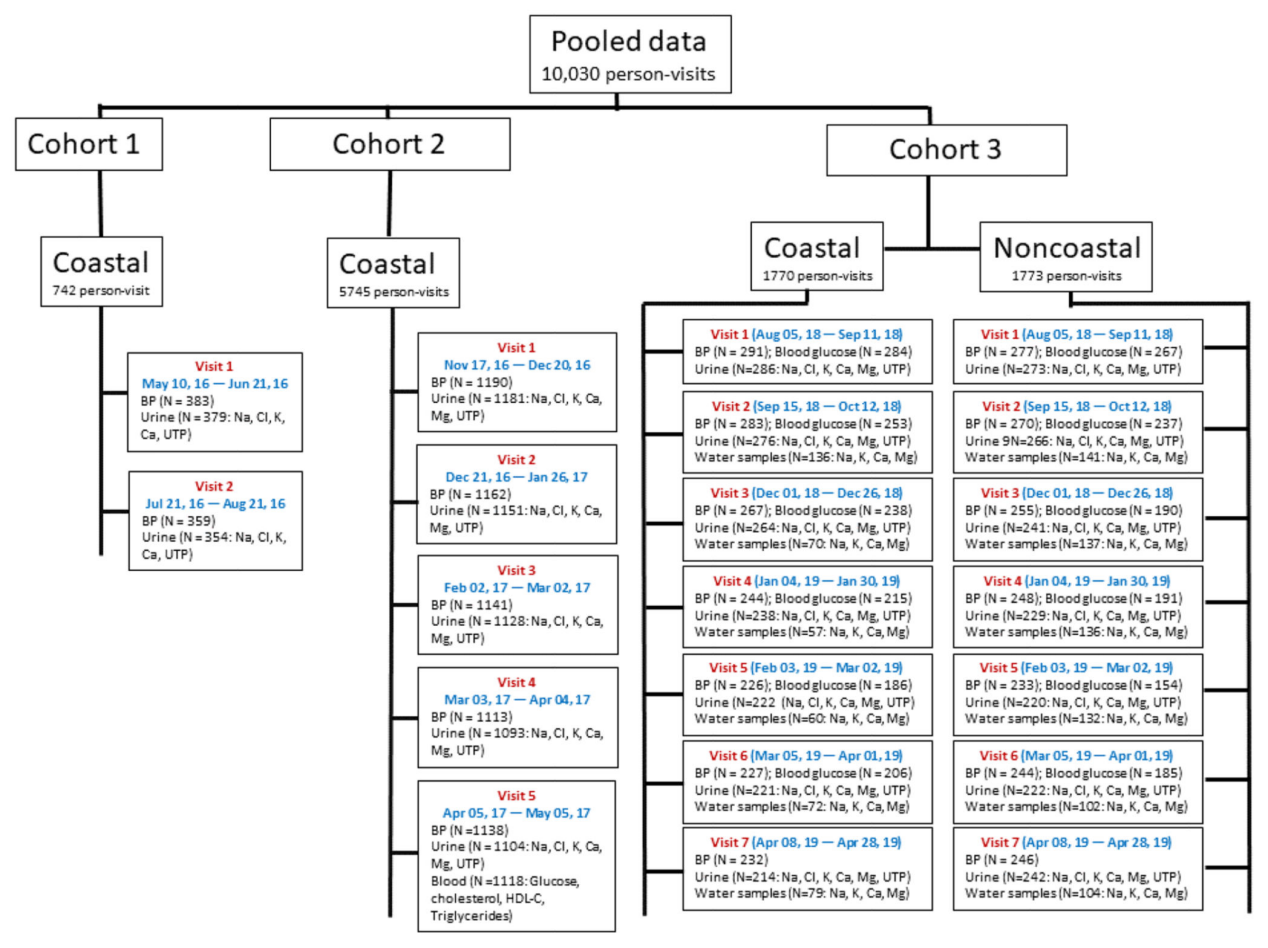
Sources of data included in analyses. BP blood pressure, Na sodium, K potassium, Ca calcium, Mg magnesium, Cl chloride, UTP urine total protein, HDL-C high-density lipoprotein cholesterol.

We included 10,034 person-visit data from three cohort studies in Bangladesh (Fig. 2), all of which were conducted by the International Centre for Diarrhoeal Disease Research, Bangladesh (icddr,b). The first cohort was a pilot study conducted in the 2016 rainy season that followed 383 participants for two visits (742 person-visits)^47^. The second study was a population-based, stepped-wedge design, randomized controlled trial that followed up 1190 participants in 16 communities for five monthly visits (5745 person-visits) during the dry season of 2017 to investigate the health effects of water access from managed aquifer recharge systems^48^. Managed aquifer recharge systems are hydrogeological interventions to reduce aquifer salinity by infiltrating rainwater and pondwater to the aquifer, and communities were randomly assigned to have access to managed aquifer recharge water^49^. The pooled analyses from the pilot and stepped-wedge trial suggested that mild-salinity water drinkers had lower mean blood pressure and higher intake of Ca and Mg compared with the freshwater drinkers^32^, which highlighted the need for understanding whether drinking saline water was the source of salubrious minerals. Therefore, a third cohort study was conducted to measure Na, K, Ca and Mg concentrations in drinking water sources longitudinally during the wet season of 2018 and the dry season of 2019 in southwest coastal and a noncoastal region. The non-coastal region was located in central Bangladesh and was not affected by saltwater intrusion from seawater, and so was included as a control hydrogeological setting with lower salinity. The third cohort study followed 293 participants from the coastal and 277 from the non-coastal region for seven visits (1773 person-visits from the coastal and 1774 from the non-coastal region).

### Drinking water sources and mineral concentration data

Participants reported their contemporary drinking water sources at all study visits. In total, 580 person-visits from three cohort studies reported drinking water from multiple sources or did not report any drinking water sources—they were excluded from analyses. We measured drinking water minerals in the 2nd to 7th visit of the third cohort study (Fig. 2). We collected households’ stored water samples, both in coastal and noncoastal regions, and sent the water samples to the Department of Agricultural Chemistry, Patuakhali Science and Technology University, for measurement of Na, K, Ca and Mg. Water samples were filtered through Whatman No. 42 filtered paper and transferred to the 50-mL conical propylene Falcon tube (Thermo Fisher Scientific, USA). After filtration, the pH of the water samples was maintained at <2.0 by adding nitric acid, and samples were preserved at 1–4 °C for further dissolved Na, K, Ca and Mg analysis^50^. Na and K were determined using the flame emission spectrophotometer (Spectrolab, UK) by selecting appropriate filters; Ca and Mg were determined by the atomic absorption spectrophotometer (AAS-Varian 55B, Australia)^51^. The respective metal standard, blank, triplicate and continuing calibration verification were included in each batch throughout the elemental analysis. Water samples were collected from one household when a cluster of households in a village shared the same drinking water source (e.g., in coastal communities). Households in the non-coastal region did not share drinking water sources. All non-coastal households in this cohort relied on groundwater in all seasons.

### Twenty-four-hour mineral intake and total urinary protein data

In all visits of the three studies, we collected participants’ 24-h urine. Participants received a 4-L plastic container for 24-hr urine collection, and a plastic mug to transfer voided urine to the 4-L plastic container. Trained field research assistants instructed participants to discard their first-morning urine and to begin 24-hurine collection by transferring the second-morning void, and then to transfer all voids of the day and night, including the next morning’s first void to the 4-L plastic container.

Field research assistants recorded the volume of the 24-h urine and took a 15-ml sample from the 4-L plastic container after stirring. They transported all urine samples to a field laboratory at 2–8 °C for processing and analysis. Urine Na, K, Ca, chloride and total protein were measured in all visits’ water samples, but urine Mg was measured during all visits of the stepped-wedge trial and third cohort study. Direct ion-selective electrode (ISE) methods^24^ were used to measure the urinary Na, K and chloride with a semi-auto electrolyte analyzer (Biolyte2000, Bio-care Corporation, Taiwan, coefficient of variation: ±5%). Urinary Ca and Mg were measured by photometric titration, and total protein was measured via the colorimetric method using a semi-auto biochemistry analyzer (Evolution 3000, BSI, Italy, coefficient of variation: <1%).

### Blood pressure, fasting blood glucose and lipid data

Blood pressure of the participants was measured in all visits using an Omron^®^ HEM-907 (accuracy: within ±4 mm Hg, Kyoto, Japan) digital monitor. Participants were advised not to consume caffeine (e.g., tea and coffee), nor to eat, smoke or perform heavy physical activities 30 min prior to measuring blood pressure. Participants rested for at least 5 min on a chair in an arm-supported sitting position before blood pressure measurement. Blood pressure was measured three times while sitting. The arithmetic mean of the three blood pressure measures was used for data analyses.

Participants’ fasting blood was collected during the 5th visit of the stepped-wedge trial, and during the 1st to 6th visits of the third cohort study. Trained phlebotomists collected 5 ml of fasting blood using aseptic precautions. Blood samples were transferred to a field laboratory for centrifugation at around 894 × *g* relative centrifugal force for 15 min at ambient temperature for plasma separation; aliquots were then stored in a −20 °C freezer. Blood glucose was measured in all blood samples using the hexokinase method^52^. Blood lipids were measured only for the 5th-visit samples of the stepped-wedge trial. Total cholesterol was measured by the enzymatic endpoint method^53^; high-density lipoprotein cholesterol (HDL-C) was measured by the direct clearance method^54^; triglycerides were measured by the enzymatic colorimetric method^55^.

### Cardiometabolic risk factor data

We recorded demographic (age, sex and religion) and socioeconomic (e.g., household asset) information on the participants and measured their anthropometric characteristics (i.e., height, weight and waist circumference). Height was measured once for each participant, but the weight was measured during each visit. We also collected data on smoking, work-related physical exercise, alcohol consumption, sleep hours and participants’ consumption of additional table salt with food during all visits. We calculated households’ wealth scores using principal component analysis of household assets, including ownership of a refrigerator, television, mobile phone, motorcycle, bicycle, sewing machine, chair, table, wrist-watch, wardrobe, wooden cot, motor pump, rice-husking machine, motorized rickshaw, car and access to electricity. We then categorized wealth scores into wealth quintiles.

### Statistical analyses

#### Descriptive statistics

We plotted histograms and kernel-density plots of 24-h urinary concentrations of all minerals and cardiometabolic biomarkers. We calculated the mean and 95% confidence interval (CI) of all minerals and cardiometabolic biomarkers that approximated a normal distribution, and the median and interquartile range (IQR) for skewed outcomes. Participants who reported the use of multiple water sources or did not report using any of the selected drinking water sources were excluded from analyses (*N* = 580 person-visits). We created box plots of mineral concentrations in each drinking water source of the third cohort study.

#### Urinary minerals and cardiometabolic biomarkers among rainwater drinkers

**Fig. 3:**
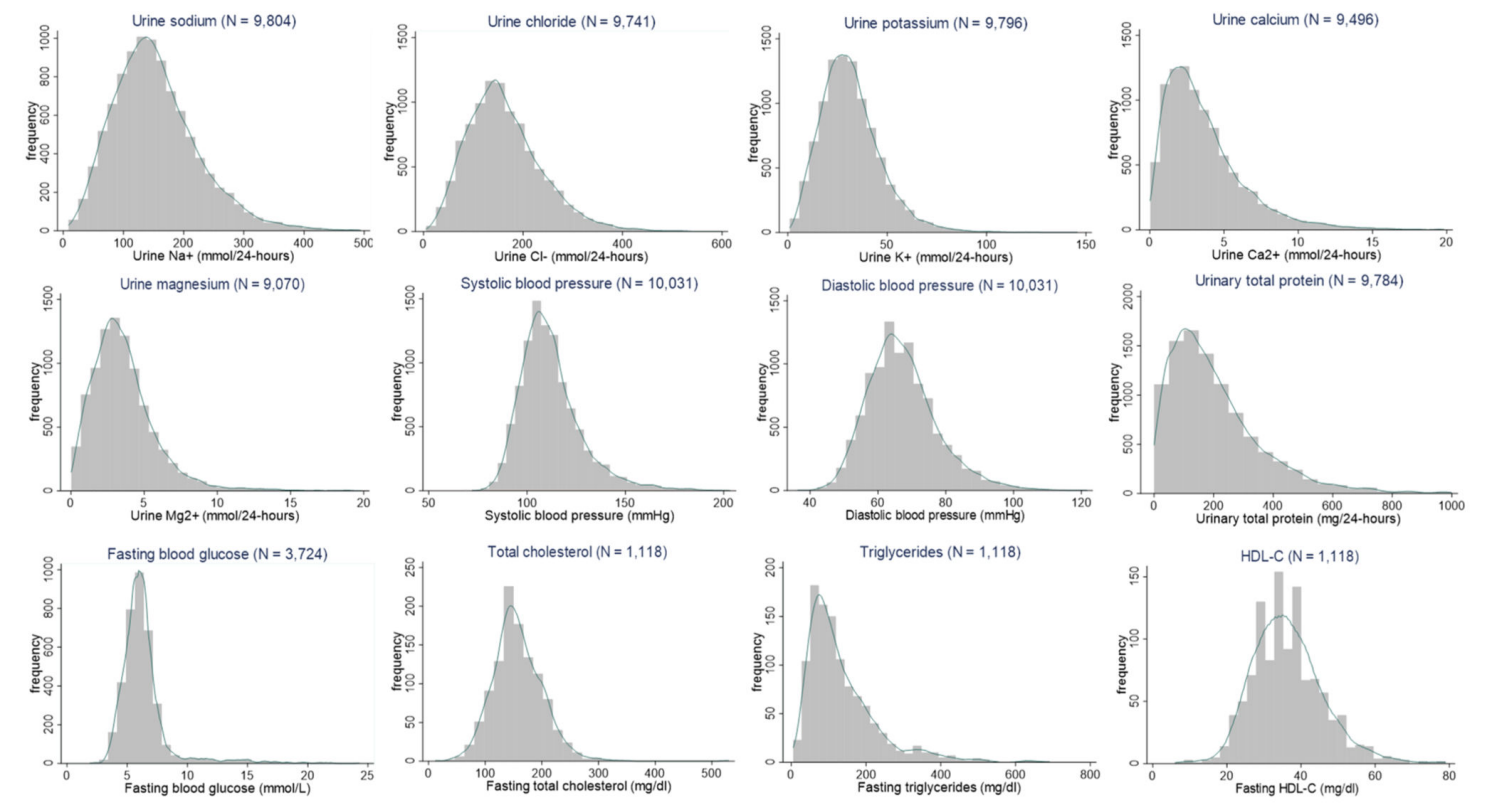
Histograms and kernel-density plots of 24-h urinary minerals and cardiometabolic markers. HDL-C high-density lipoprotein cholesterol.

We used multilevel models to contrast the levels of 24-h urinary minerals and cardiometabolic biomarkers between persons drinking rainwater and persons drinking water from alternative water sources. We considered three alternative water sources: (1) pondwater, (2) coastal groundwater and (3) combined groundwater (both coastal and non-coastal). We used multilevel linear regression models for all 24-h urinary Na, chloride, K, Mg, SBP, DBP, FBG, total cholesterol and HDL-C. Since 24-h urinary Ca, urinary total protein and fasting plasma triglycerides had skewed distributions (Fig. 3), we used multilevel gamma regression models^56^ to estimate the ratio of median biomarkers between rainwater drinkers and drinkers of alternative water sources. The models included three random intercepts accounting for the nesting of longitudinal visits within participants, participants within households and households within communities. Since fasting plasma total cholesterol, HDL-C and triglycerides were measured only during the 5th visit of the stepped-wedge trial, the multilevel models for those outcomes had two random intercepts: for clustering of individuals within households, and households within communities.

#### Propensity-score-matched analyses

Several covariates were unequally distributed across the person-visits of different water sources. Therefore, we used propensity-score matching of person-visits of rainwater drinkers to reference water sources. The propensity-score model was conditioned on age, sex, BMI, smoking, alcohol consumption, physical activity, religion, sleep hours, consumption of table salt and household wealth using nearest-neighbour matching by Mahalanobis distance^57^. Of the cardiometabolic biomarkers, blood pressure and urine total protein were measured in all visits, fasting blood glucose was measured in the fifth visit of cohort 2 and the first six visits of cohort 3 studies and fasting blood lipids were measured only in the fifth visit of the cohort 2 study. Therefore, 1598 person-visits of rainwater drinkers were matched with 1598 person-visits of coastal groundwater drinkers for blood pressure and urine total protein, 716 person-visits of rainwater drinkers were matched with 716 personvisits of coastal groundwater drinkers for FBG and 100 person-visits of rainwater drinkers were matched with 100 person-visits of coastal groundwater drinkers for blood lipids. Similarly, person-visits of pondwater and combined coastal groundwater drinkers were also matched with person-visits of rainwater drinkers for each of the biomarkers. In the propensity-score-matched subpopulation, we used similar multilevel linear or gamma regression models described above.

We estimated multilevel models using maximum likelihood and reported cluster robust standard errors considering the community as the highest level of clustering. We reported findings of unadjusted models (model 1); models adjusted for age, sex and BMI (model 2); models that additionally adjusted for participants’ smoking status, alcohol consumption, physical activity, religion, sleep duration, consumption of table salt with food and household wealth (model 3); models that additionally adjusted for dry versus wet seasons of data collection (model 4). Age and BMI were used as linear continuous variables in all models, but other covariates were used as categorical variables. We used a nominal significance P-value threshold of 0.05. We performed statistical analyses in Stata/SE, version 16.0, but propensity score matching was done using MatchIT package in R, version 3.3.0^58^.

#### Sensitivity analyses

In the sensitivity analyses, we used conditional logistic regression to model the within-person associations of drinking rainwater with minerals and cardiometabolic biomarkers. The outcome for these models was binary (drinking rainwater vs. alternative water source), and the continuous predictors were urine minerals and cardiometabolic biomarkers. Separate models were fitted for each predictor. Since these models are making within-person comparisons, they control for all timeinvariant person-level confounders through matching.

#### Multiple comparisons

The family-wise error rate was controlled for multiple comparisons through a Bonferroni correction (*α* = 0.01)^59^. The Bonferroni-adjusted *p*-value threshold accounted for 12 hypothesis tests of urine minerals and cardiometabolic biomarkers.

## Figures and Tables

**Table 1:** Characteristics of the participants across person-visits of different drinking water sources.

Characteristics	Rainwater (*N* = 1620 person-visits)	Pond water (*N* = 2836 person-visits)	Coastal groundwater (*N* = 3225 person-visits)	Non-coastal groundwater (*N* = 1773 person-visits)
Age in years, mean (95% CI)	43.5 (42.8, 44.2)	43.2 (42.7, 43.8)	42.6 (42.1, 43.1)	40.7 (40.1, 41.4)
Male sex, % (n)	43.3 (702)	39.5 (1120)	40.0 (1291)	33.8 (599)
Body mass index, mean (95% CI)	22.9 (22.8, 23.2)	22.4 (22.2, 22.5)	21.9 (21.8, 22.0)	22.8 (22.6, 23.0)
Smoking categories, % (*n*)				
Never	69.3 (1117)	59.9 (1694)	51.3 (1652)	74.2 (1304)
Former	7.8 (125)	10.2 (287)	8.5 (273)	19.4 (341)
Current	22.9 (370)	29.9 (847)	40.2 (1296)	6.4 (112)
Alcohol consumption, % (*n*)	2.1 (34)	1.9 (55)	3.5 (111)	2.8 (49)
WHO—work-related physical activity, % (*n*)				
Sedentary	44.9 (724)	49.4 (1397)	31.5 (1015)	15.0 (265)
Moderate^a^	30.3 (489)	23.9 (675)	40.7 (1311)	56.6 (996)
Vigorous^b^	24.8 (399)	26.7 (756)	27.8 (895)	28.3 (497)
Hindu religion, % (*n*)	55.3 (895)	54.1 (1533)	60.3 (1943)	10.4 (185)
Hours of sleep, % (*n*)				
<6 h	19.0 (307)	21.9 (618)	19.2 (617)	10.5 (185)
≥6 to <9 h	74.3 (1197)	64.9 (1836)	70.6 (2274)	82.9 (1457)
≥9 h	6.7 (108)	13.2 (374)	10.3 (330)	6.6 (115)
Added table salt with food, % (*n*)	49.8 (802)	60.0 (1698)	60.5 (1950)	84.1 (1478)
Household wealth index, % (*n*)				
Lowest	15.8 (256)	18.6 (524)	27.2 (876)	10.9 (193)
Second	14.9 (241)	22.9 (644)	20.0 (643)	18.3 (324)
Third	13.4 (217)	20.3 (571)	20.8 (670)	21.6 (382)
Fourth	22.6 (366)	17.7 (498)	16.2 (521)	25.9 (458)
Highest	33.3 (538)	20.5 (578)	15.8 (507)	23.4 (415)

*CI* confidence interval, *IQR* interquartile range, *WHO* World Health Organization.

a Work-related moderate-intensity activity causes a small increase in breathing or heart rate (e.g., brisk walking or carrying light loads) for at least 10 min continuously.

b Work-related vigorous-intensity activity causes a large increase in breathing or heart rate (e.g., carrying or lifting heavy loads, digging or construction work) for at least 10 min continuously.

**Table 2:** Twenty-four-hour urinary minerals and cardiometabolic biomarkers across person-visits of different drinking water sources.

Twenty-four-hour urine minerals and cardiometabolic biomarkers	Rainwater (*N* = 1620 person-visits)	Pondwater (*N* = 2836 person-visits)	Coastal groundwater (*N* = 3225 person-visits)	Non-coastal groundwater (*N* = 1773 person-visits)
Urine Na (mmol/24 hours), mean (95% CI)	138.5 (134.7, 142.3)	159.6 (157.0, 162.2)	166.5 (164.0, 169.2)	135.2 (131.6, 138.8)
Urine chloride (mmol/24 hours), mean (95% CI)	147.7 (144.2, 151.1)	171.7 (168.9, 174.5)	175.4 (172.6, 178.3)	143.8 (139.7, 148.0)
Urine K (mmol/24 hours), mean (95% CI)	31.8 (31.1, 32.6)	33.5 (32.9, 34.0)	33.3 (32.8, 33.9)	23.9 (23.3, 24.5)
Urine Ca (mmol/24 hours), median (IQR)	2.7 (1.6–4.2)	3.3 (2.0–5.0)	3.6 (2.0–5.9)	2.1 (1.3–3.5)
Urine Mg (mmol/24 hours), mean (95% CI)	3.3 (3.2, 3.4)	3.8 (3.7, 3.9)	4.0 (3.9, 4.1)	3.0 (2.9, 3.1)
SBP (mm Hg), mean (95% CI)	114.2 (113.4, 115.0)	114.0 (113.4, 114.6)	111.2(110.7, 111.8)	108.2 (107.5, 108.9)
DBP (mm Hg), mean (95% CI)	68.3 (67.8, 68.8)	68.1 (67.7, 68.5)	66.0 (65.6, 66.3)	66.6 (66.1, 67.1)
Urine total protein (mg/dl), median (IQR)	174.0 (98.6–278.9)	187.7 (102.9–320.2)	188.7 (111.4–294.5)	105.8 (55.8–177.3)
Fasting blood glucose (mmol/L), mean (95% CI)	6.6 (6.5, 6.8)	6.3 (6.2, 6.5)	5.3 (5.2, 5.5)	6.9 (6.8, 7.0)
Fasting triglycerides (mg/dl), median (IQR)	133 (87-196)	108 (72-167)	106 (69-178)	—
Fasting total cholesterol (mg/dl), mean (95% CI)	164.2 (155.9, 172.5)	145.4 (140.5, 150.2)	161.9 (158.3, 165.5)	—
HDL-C (mg/dl), mean (95% CI)	34.1 (32.6, 35.7)	37.3 (36.2, 38.4)	36.2 (35.5, 37.0)	—

“—” biomarker not measured for non-coastal groundwater drinkers. *CI* confidence interval, *IQR* interquartile range, SBP systolic blood pressure, *DBP* diastolic blood pressure, *HLD-C* high-density lipoprotein cholesterol.

**Table 3:** Daily mineral intakes measured as 24-h urinary mineral concentrations, and cardiometabolic biomarkers among the rainwater drinkers compared with coastal groundwater drinkers.

	Model 1	Model 2	Model 3	Model 4^a^
Twenty-four-hour urinary Na (mmol/24 h)	−17.86 (−24.55, −11.16)	−17.84 (−24.80, −10.89)	−16.46 (−22.76, −10.16)	−13.42 (−18.27, −8.57)
Twenty-four-hour urinary Cl (mmol/24 h)	−18.82 (−26.33, −11.30)	−19.15 (−26.80, −11.50)	−17.64 (−24.60, −10.69)	−13.44 (−19.21, −7.66)
Twenty-four-hour urinary K	−2.52 (−3.90, −1.14)	−2.70 (−4.18, −1.22)	−2.62 (−4.04, −1.20)	−2.00 (−3.16, −0.85)
Twenty-four-hour urinary Ca^b^	0.69 (0.63, 0.77)	0.69 (0.62, 0.76)	0.69 (0.63, 0.77)	0.72 (0.64, 0.80)
Twenty-four-hour urinary Mg (mmol/24 h)	−0.77 (−1.22, −0.32)	−0.77 (−1.20, −0.35)	−0.74 (−1.17, −0.31)	−0.57 (−1.02, −0.16)
Systolic blood pressure (mm Hg)	1.84 (0.78, 2.90)	1.59 (0.55, 2.62)	1.65 (0.59, 2.70)	2.15 (1.02, 3.27)
Diastolic blood pressure (mm Hg)	1.46 (0.78, 2.14)	1.37 (0.71, 2.02)	1.40 (0.77, 2.03)	1.82 (1.19, 2.45)
Twenty-four-hour urinary total protein^b^	1.13 (1.00, 1.28)	1.10 (0.97, 1.26)	1.12 (1.00, 1.26)	1.16 (1.01, 1.33)
Fasting blood glucose (mmol/L)	0.90 (0.60, 1.21)	0.81 (0.48, 1.14)	0.67 (0.32, 1.02)	0.59 (0.17, 1.01)
Fasting total cholesterol (mg/dl)	−0.38 (−8.59, 7.82)	−1.38 (−10.88, 8.11)	−2.30 (−12.92, 8.32)	---
HDL-C (mg/dl)	−2.15 (−4.92, 0.62)	−1.73 (−4.37, 0.90)	−2.02 (−5.85, 0.81)	---
Fasting triglycerides^b^	1.12 (0.98, 1.27)	1.07 (0.92, 1.25)	1.06 (0.89, 1.25)	---

“---” lipids were not adjusted for seasonality since lipids were measured only in the fifth visits of the cohort 2 study. *HDL-C* high-density lipoprotein cholesterol. Model 1: unadjusted. Model 2: adjusted for age, sex and body mass index. Model 3: additionally adjusted for smoking, alcohol consumption, physical activity, religion, sleep hours, consumption of table salt with food and household wealth.

a Model 4 additionally adjusted for seasonality.

b Refers to the ratio of medians of 24-h urinary minerals or biomarkers between rainwater drinkers and the reference group.

**Table 4:** Daily mineral intakes measured as 24-h urinary mineral concentrations, and cardiometabolic biomarkers among the rainwater drinkers compared with groundwater drinkers (both coastal and non-coastal).

	Model 1	Model 2	Model 3	Model 4^a^
Twenty-four-hour urinary Na (mmol/24 h)	−16.0 (−22.8, −9.2)	−15.74 (−22.85, −8.63)	−14.57 (−21.16, −7.97)	−12.57 (−18.55, −6.59)
Twenty-four-hour urinary Cl (mmol/24 h)	−17.67 (−25.13, −10.21)	−17.90 (−25.53, −10.28)	−16.47 (−23.52, −9.42)	−13.61 (−19.94, −7.28)
Twenty-four-hour urinary K (mmol/24 h)	−2.16 (−3.71, −0.60)	−2.27 (−3.92, −0.61)	−2.13 (−3.73, −0.53)	−2.00 (−3.50, −0.50)
Twenty-four-hour urinary Ca^b^	0.70 (0.62, 0.78)	0.70 (0.62, 0.78)	0.70 (0.62, 0.78)	0.72 (0.64, 0.80)
Twenty-four-hour urinary Mg (mmol/24 h)	−0.68 (−1.14, −0.22)	−0.68 (−1.11, −0.25)	−0.66 (−1.08, −0.23)	−0.46 (−0.90, −0.02)
Systolic blood pressure (mm Hg)	1.92 (0.88, 2.96)	1.65 (0.65, 2.65)	1.72 (0.70, 2.74)	2.35 (1.29, 3.41)
Diastolic blood pressure (mm Hg)	1.43 (0.76, 2.10)	1.32 (0.67, 1.97)	1.37 (0.74, 1.99)	1.96 (1.38, 2.53)
Twenty-four-hour urinary total protein^b^	1.15 (1.02, 1.30)	1.12 (0.99, 1.28)	1.14 (1.01, 1.24)	1.12 (0.99, 1.27)
Fasting blood glucose (mmol/L)	0.83 (0.50, 1.15)	0.73 (0.39, 1.06)	0.65 (0.31, 0.99)	0.73 (0.38, 1.07)

Model 1: unadjusted. Model 2: adjusted for age, sex and body mass index. Model 3: additionally adjusted for smoking, alcohol consumption, physical activity, religion, sleep hours, consumption of table salt with food and household wealth.

a Model 4 additionally adjusted for seasonality.

b Refers to the ratio of medians of 24-h urinary minerals or biomarkers between rainwater drinkers and the reference group.

**Table 5:** Daily mineral intakes measured as 24-h urinary mineral concentrations, and cardiometabolic biomarkers among the rainwater drinkers compared with pond water drinkers.

	Model 1	Model 2	Model 3	Model 4^a^
Twenty-four-hour urinary Na (mmol/24 h)	−20.01 (−26.85, −13.17)	−19.72 (−26.60, 12.83)	−16.91 (−22.88, −10.94)	−12.09 (−18.50, −5.68)
Twenty-four-hour urinary Cl (mmol/24 h)	−23.34 (−30.93, −15.75)	−23.45 (−31.29, −15.62)	−20.32 (−26.97, −13.67)	−12.95 (−19.82, −6.09)
Twenty-four-hour urinary K (mmol/24 h)	−3.33 (−5.19, −1.48)	−3.41 (−5.26, −1.56)	−3.25 (−4.97, −1.54)	−2.23 (−3.92, −0.53)
Twenty-four-hour urinary Ca^b^	0.80 (0.73, 0.87)	0.80 (0.73, 0.87)	0.81 (0.75, 0.88)	0.86 (0.79, 0.93)
Twenty-four-hour urinary Mg (mmol/24 h)	−0.47 (−0.82, −0.11)	−0.47 (−0.82, −0.12)	−0.46 (−0.77, −0.15)	−0.17 (−0.45, 0.11)
Systolic blood pressure (mm Hg)	−0.61 (−1.89, 0.66)	−1.07 (−2.23, 0.09)	−1.04 (−2.45, 0.37)	0.91 (−0.54, 2.36)
Diastolic blood pressure (mm Hg)	−0.62 (−1.48, 0.24)	−0.79 (−1.60, 0.02)	−0.88 (−1.73, −0.04)	0.53 (−0.39, 1.44)
Twenty-four-hour urinary total protein^b^	0.82 (0.70, 0.95)	0.81 (0.69, 0.96)	0.86 (0.75, 0.98)	0.89 (0.74, 1.07)
Fasting blood glucose (mmol/L)	0.09 (−0.12, 0.30)	0.08 (−0.13, 0.27)	0.03 (−0.19, 0.25)	−0.04 (−0.21, 0.12)
Fasting total cholesterol (mg/dl)	0.69 (−10.48, 11.85)	−0.02 (−11.65, 11.61)	−1.18 (−15.19, 12.83)	---
HDL-C (mg/dl)	−3.39 (−6.16, −0.62)	−2.95 (−5.81, −0.08)	−3.40 (−6.69, −0.11)	---
Fasting triglycerides^b^	1.17 (1.00, 1.38)	1.14 (0.97, 1.36)	1.10 (0.89, 1.35)	---

*“---”* lipids were not adjusted for seasonality since lipids were measured only in the fifth visits of the cohort 2 study. *HDL-C* high-density lipoprotein cholesterol. Model 1: unadjusted. Model 2: adjusted for age, sex and body mass index. Model 3: additionally adjusted for smoking, alcohol consumption, physical activity, religion, sleep hours, consumption of table salt with food and household wealth.

a Model 4 additionally adjusted for seasonality.

b Refers to the ratio of medians of 24-h urinary minerals or biomarkers between rainwater drinkers and the reference group.

**Table 6:** Twenty-four-hour urinary mineral concentrations, and cardiometabolic biomarkers among the rainwater drinkers compared with the groundwater and pond water drinkers in propensity-score-matched analyses.

Biomarkers	Model 1	Model 2	Model 3	Model 4^a^
Coastal groundwater drinkers considered as the comparison group				
SBP (mm Hg)	1.39 (0.39, 2.38)	1.08 (0.11, 2.05)	1.20 (0.20, 2.20)	1.74 (0.71, 2.76)
DBP (mm Hg)	1.25 (0.62, 1.89)	1.08 (0.43, 1.73)	1.18 (0.53, 1.82)	1.61 (1.03, 2.19)
Twenty-four-hour urinary total protein^b^	1.13 (1.00, 1.28)	1.13 (1.00, 1.28)	1.13 (1.01, 1.27)	1.17 (1.02, 1.33)
FBG (mmol/L)	0.84 (0.55, 1.13)	0.81 (0.47, 1.14)	0.67 (0.32, 1.02)	0.59 (0.17, 1.01)
Fasting total cholesterol (mg/dl)	−1.94 (−11.11, 7.29)	−3.79 (−13.42, 5.84)	−2.09 (−13.07, 8.89)	---
HDL-C (mg/dl)	−3.22 (−6.78, 0.35)	−2.91 (−6.14,0.33)	−3.25 (−6.35, −0.15)	---
Fasting triglycerides^b^	1.20 (1.00, 1.45)	1.16 (0.97, 1.39)	1.16 (1.00, 1.35)	---
Pond water drinkers considered as the comparison group				
SBP (mm Hg)	−0.64 (−2.00, 0.71)	−1.08 (−2.29, 0.12)	−1.11 (−2.44,0.23)	0.81 (−0.42, 2.03)
DBP (mm Hg)	−0.84 (−1.89, 0.19)	−1.03 (−1.97, −0.09)	−1.08 (−2.04, −0.11)	0.35 (−0.58, 1.28)
Twenty-four-hour urinary total protein^b^	0.85 (0.73, 0.98)	0.85 (0.73, 0.98)	0.88 (0.76, 1.01)	0.92 (0.76, 1.10)
FBG (mmol/L)	0.05 (−0.18, 0.27)	0.03 (−0.18, 0.24)	0.01 (−0.21, 0.23)	−0.04 (−0.22, 0.14)
Fasting total cholesterol (mg/dl)	12.63 (−7.98, 33.25)	10.93 (−9.06, 30.93)	10.27 (−8.70, 29.25)	---
HDL-C (mg/dl)	−2.86 (−5.64, −0.07)	−2.84 (−5.75, 0.08)	−3.29 (−7.08, 0.51)	---
Fasting triglycerides^b^	1.19 (0.97, 1.45)	1.19 (0.98, 1.45)	1.20 (1.02, 1.42)	---

“---” lipids were not adjusted for seasonality since lipids were measured only in the fifth visits of the cohort 2 study. *SBP* systolic blood pressure, *DBP* diastolic blood pressure, *FBG* fasting blood glucose, *HDL-C* high-density lipoprotein cholesterol. Model 1: unadjusted. Model 2: adjusted for age, sex and body mass index. Model 3: additionally adjusted for smoking, alcohol consumption, physical activity, religion, sleep hours, consumption of table salt with food and household wealth.

a Model 4 additionally adjusted for seasonality.

b Refers to the ratio of medians of 24-h urinary minerals or biomarkers between rainwater drinkers and the reference group.

**Table 7:** Within-person effect of drinking rainwater on 24-h urinary mineral concentrations and cardiometabolic biomarkers among conditional logistic regression models.

Minerals and cardiometabolic biomarkers	Matched person-visits of pond water drinkers considered as comparison group			Matched person-visits of coastal groundwater drinkers considered as comparison group		
	Odds ratio	95% CI	*p*-value	Odds ratio	95% CI	*p*-value
Urinary Na	0.993	0.991, 0.995	<0.001	0.996	0.994, 0.998	<0.001
Urinary chloride	0.992	0.990, 0.994	<0.001	0.994	0.992, 0.996	<0.001
Urinary K	0.971	0.962, 0.980	<0.001	0.980	0.972, 0.989	<0.001
Urinary Ca	0.788	0.741, 0.837	<0.001	0.719	0.678, 0.763	<0.001
Urinary Mg	0.866	0.816, 0.918	<0.001	0.832	0.783, 0.883	<0.001
Systolic blood pressure	0.990	0.979, 1.002	0.113	1.034	1.020, 1.049	<0.001
Diastolic blood pressure	0.981	0.963, 0.998	0.025	1.055	1.034, 1.077	<0.001
Twenty-four-hour urinary total protein	1.000	1.000, 1.000	0.246	1.000	0.999, 1.000	<0.001
Fasting blood glucose	1.023	0.893, 1.172	0.746	2.830	2.009, 3.987	<0.001

Non-coastal groundwater could not be included for within-person effect of drinking rainwater since none of the participants reported drinking rainwater in the non-coastal region. Within-person effect of drinking rainwater on lipids could not be determined since lipids were measured only in one visit.

## Data Availability

The data that support the findings of this study are available from the corresponding author upon reasonable request.
